# Cost-effectiveness of the PDSAFE personalised physiotherapy intervention for fall prevention in Parkinson’s: an economic evaluation alongside a randomised controlled trial

**DOI:** 10.1186/s12883-020-01852-8

**Published:** 2020-08-11

**Authors:** Yiqiao Xin, Ann Ashburn, Ruth M. Pickering, Kim Chivers Seymour, Sophia Hulbert, Carolyn Fitton, Dorit Kunkel, Ioana Marian, Helen C. Roberts, Sarah E. Lamb, Victoria A. Goodwin, Lynn Rochester, Emma McIntosh, on behalf of the PDSAFE Collaborative group, A. Ashburn, K. Seymour, H. C. Roberts, R. M. Pickering, S. Lamb, C. Ballinger, V. A. Goodwin, L. Rochester, A. Nieuwboer, E. McIntosh, D. Kunkel, S. Hulbert, C. Fitton, I. Marian, Y. Xin, A. Rowsell, R. Summer

**Affiliations:** 1 Health Economics and Health Technology Assessment (HEHTA), Institute of Health & Wellbeing, University of Glasgow, 1 Lilybank gardens, Glasgow, G12 8RZ UK; 2 School of Health Science, University of Southampton, Southampton, UK; 3 Faculty of Medicine, University of Southampton, Southampton, UK; 4 Centre for Statistics in Medicine, Oxford Clinical Trials Research Unit (OCTRU), University of Oxford, Oxford, UK; 5 College of Medicine and Health, University of Exeter, Exeter, UK; 6 Clinical Ageing Research Unit, Institute of Neuroscience, Newcastle University, Newcastle Upon Tyne, UK

**Keywords:** Parkinson’s, Physiotherapist, Cost-effectiveness, Quality of life, Cost

## Abstract

**Background:**

PDSAFE is an individually-tailored, physiotherapist-delivered, balance, strength and strategy training programme aimed at preventing falls among people with Parkinson’s. We evaluated the cost-effectiveness of PDSAFE compared with usual care for people with Parkinson’s at higher risk of falling, from a UK National Health Service and Personal Social Service perspective.

**Methods:**

Resource use and quality of life data (EQ-5D-3L) were collected from 238 participants randomised to the PDSAFE intervention and 236 participants randomised to control, at baseline, 3 months, 6 months (primary outcome), and 12 months. Adjusted cost and quality-adjusted life-years (QALYs) were estimated using generalised linear models and uncertainty estimated using a non-parametric bootstrap.

**Results:**

Over 6 months, the PDSAFE intervention was associated with an incremental cost of £925 (95% CI £428 to £1422) and a very small and statistically insignificant QALY gain of 0.008 (95% CI − 0.006 to 0.021). The resulting incremental cost-effectiveness ratio (ICER) was £120,659 per QALY and the probability of the intervention being cost-effective at a UK threshold of £30,000/QALY was less than 1%. The ICER varied substantially across subgroups although no subgroup had an ICER lower than the £30,000 threshold. The result was sensitive to the time horizon with the ICER reducing to £55,176 per QALY when adopting a 12-month time horizon and assuming a sustained treatment effect on QoL, nevertheless, the intervention was still not cost-effective according to the current UK threshold.

**Conclusions:**

Evidence from this trial suggests that the PDSAFE intervention is unlikely to be cost-effective at 6 months. The 12-month analysis suggested that the intervention became closer to being cost-effective if quality of life effects were sustained beyond the intervention period, however this would require confirmation. Further research, including qualitative studies, should be conducted to better understand the treatment effect of physiotherapy and its impact on quality of life in people with Parkinson’s given existing mixed evidence on this topic.

**Trial registration:**

ISRCTN48152791. Registered 17 April 2014. http://www.isrctn.com/ISRCTN48152791

## Background

Improving balance and reducing falls are identified as the leading priority of the James Lind Alliance [1] top 10 priorities for people with Parkinson’s [2]. People with Parkinson’s are twice as likely to fall as healthy older people; a recent US study revealed that the overall falls incidence rate ratio comparing people with Parkinson’s versus non-Parkinson’s subjects was 2.05 (95% confidence interval (CI) 1.88–2.24), adjusting for comorbidity and medications [3]. Falling can have serious health and quality of life consequences such as fractures, [4, 5] immobility and fear of falling [6] leading to dependency and social isolation [7]. Falling also increases the psychological burden on informal caregivers, increases personal costs (e.g. home alteration), [8] as well as costs to the health care system. A recently published (2018) 10-year UK cohort study analysed linked health care data from 7060 people with Parkinson’s, and the cost of inpatient care was found to be £900 per year greater compared to age and sex matched controls [9]. Among the reasons for admission to inpatient care (e.g. infection, dementia etc.), falls and related fractures were most frequently cited, accounting for 13% of the total cases [10]. In Germany, direct costs among the people with Parkinson’s who experienced falls was considerably higher than among people with Parkinson’s who did not fall (€3180 and €1300 respectively) due to the longer duration of hospitalisation [11].

Frequent and repeated falls are not prevented with medication, [12] however, several studies have shown that physiotherapy can be beneficial through exercise, strength and balance training, [13–15] yet it is unclear whether physiotherapy reduces the rate of falling [16, 17]. A UK multi-centre, single blind, randomised controlled trial, was conducted to evaluate the effectiveness and cost-effectiveness of a novel, home-based physiotherapy programme, PDSAFE, compared to usual care for falls management in people with Parkinson’s [18].

Economic evaluation alongside clinical trials compares the cost and outcomes of health care interventions implemented in the randomised arm of the trial and makes recommendations to decision makers regarding intervention cost-effectiveness. The objective of this study was to estimate the cost-effectiveness of the PDSAFE intervention compared to usual care in people with Parkinson’s who had a history of falling.

## Methods

### Trial eligibility criteria, baseline characteristics, intervention, and outcome measures

Between 2014 and 2016, 474 participants were randomised to receive the PDSAFE intervention (*n* = 238) or usual care (*n* = 236) for a six-month period. Patients were eligible if they: had a confirmed consultant’s diagnosis of Parkinson’s; were independently mobile; experienced ≥1 fall in the previous 12 months; scored ≥24 on Mini-Mental State Examination [19]; and were able to give informed consent, understand and follow commands, and complete a guided personalised exercise and strategy programme. These participants were on average aged 71 (SD 7.7) and 73 (SD 7.7) in the intervention and control group, with a mean of 8 (SD 6.6) and 8 (SD 5.8) years of diagnosis of Parkinson’s respectively, a median Hoehn & Yahr scale stage of 3 in both groups, and a mean Movement Disorder Unified Parkinson’s Disease Rating Scale (UPDRS) score of 32 (SD 15.2) and 33 (SD 17.3) respectively [18]. The proportion of participants that had freezing of gait in the past month prior to randomisation was 64% in the intervention group and 59% in the control group. Both of the groups had a median of 3 falls in the 12 months prior to screening, and 78% in the intervention group and 80% in the control group had repeat falling during the same period [18]. In the 3 months prior to randomisation, the intervention and control participants had a median of 2 and 1 fall respectively [18]. The trial protocol, including a summary of economic evaluation methods, and the detailed trial primary outcome results are published elsewhere [14, 18, 20].

The PDSAFE programme was personalised to each participant by a physiotherapist through a clinical assessment and targeted specific problems in their home environment. Exercises and strategies were selected from a menu of activities and provided to participants in a booklet accompanied by video vignettes on a DVD. Each participant was provided up to 12 one-hour sessions. Participants in the control group received usual care plus a DVD on Parkinson’s and one advice session after trial completion. All participants were assumed on appropriate doses of medications during the trial following specialist Parkinson’s services. The primary outcome was the risk of repeat falling during the 6 months post randomisation.

### Overview of economic evaluation methods

A ‘within-trial’ 6-month cost-utility analysis was conducted from the perspective of UK National Health Service (NHS) and Personal Social Service (PSS) under the principle of intention-to-treat, following recommendations for good practice [21, 22]. Costs included intervention costs and NHS and social care cost. Resource use, and QoL measured using the EQ-5D-3L instrument [23] were collected at baseline, 3 and 6 months. Missing data were imputed using multiple imputation methods [24]. The incremental cost-effectiveness ratio (ICER) was compared with a £20,000–£30,000 per QALY threshold currently applied by UK's NICE [21]. Subgroup and sensitivity analyses in relation to cost reduction strategies and longer follow-up period up to 12 months were conducted. A non-parametric bootstrap with 1000 iterations was conducted to explore the uncertainty around the ICER estimate. All analyses were undertaken in STATA/SE 12.0 [24]. Details of methods were previously published elsewhere [25].

### Routine resource use

Questionnaires were posted to participants to collect NHS and social care resource use data. These included: i) primary care services (GP visits, routine physiotherapy, etc.), ii) secondary care services (ambulance, hospitalisation, etc.), iii) social care services (home care visits, meals on wheels, etc.), and iv) medication use. Medication was not included in the total costs given the low possibility that the intervention would impact on the consumption of medication, as well as the large number of low-cost medications reported. Unit costs were sourced from the Personal Social Service Research Unit (PSSRU) [26] and the NHS National Schedule of Reference Costs [27] for a base financial year 2016 (Supplementary material 1).

### Cost of personalised home-based physiotherapy

The cost of therapy sessions included physiotherapists’ salaries, training, travel, equipment and consumables. Physiotherapists’ salaries were estimated based on the UK NHS band six full-time equivalent. Physiotherapists received 2 days of training, costed as the time spent by the trainer (lead physiotherapist), room hire and training materials. Equipment included printed materials and DVDs of the intervention demonstration provided to each participant in the intervention group, and a variety of assisted equipment provided to a proportion of participants as needed, including weighted vests, balance pads, and steps. All equipment was retained by the physiotherapists and used across participants. Costs associated with the equipment were annualised with a discount rate of 3.5% [21] over the anticipated life span where appropriate.

### Quality of Life (QOL) and Quality Adjusted Life Years (QALYs)

QoL was measured using the EQ-5D-3L [28] instrument and utility scores were generated using tariff values previously elicited from a UK general population sample [29]. The area-under-the-curve approach was used to estimate QALYs where the change between the two assessment points was assumed to be linear [30]. Participants who died had their utility score and resource use set to zero from the next assessment point after death.

### Missing data and multiple imputation

Missing cost or EQ-5D-3L utility values were imputed using multiple imputation with chained equations and the predictive mean matching method was used [24]. The imputation was conducted separately within the PDSAFE and control groups. 50 imputed datasets were created. EQ-5D-3L was imputed at the utility value level (rather than at the dimension level), and cost items were imputed as categories (NHS cost excluding hospitalisation, hospitalisation cost, and social care cost) following recommended practice [24]. Missingness at item level was imputed with zero assuming no resource was used if any other item within the same cost categories was completed in the questionnaire. Given the relatively high cost of hospitalisation, any missing data in hospitalisation were cross checked with falls diaries before imputation for accuracy purposes.

### Statistical analysis

Adjusted differences in costs and QALYs between the randomised groups were estimated using generalised linear models. Cost and QALYs were estimated using a Gamma distribution and a normal distribution respectively. Baseline utility scores and costs were adjusted. Other covariates adjusted were selected based on statistical significance in a regression predicting the likelihood of missingness from the full list of demographics, medical history, and screening measures. The resulted list of covariates included: age, gender, Hoehn & Yahr scale, [31] Montreal Cognitive assessment score, [32] Mini-Mental State Examination score, [19] presence of diabetes, history of myocardial infarction, history of ischemic heart disease, history of deep brain stimulation and presence of an informal carer.

The method of recycled predictions was used to estimate mean costs and QALYs of each trial arm. The ICER was estimated by dividing the adjusted cost difference between the randomised groups by the adjusted QALY difference. Uncertainty in the ICER estimate was evaluated using a 1000-iteration bootstrap and the probabilities that the intervention was cost-effective under a wide range of hypothetical thresholds (£0 - £200,000) were presented in the cost- effectiveness acceptability curve.

### Sensitivity analysis

A variety of sensitivity analyses were conducted to assess the impact on the cost-effectiveness results. First, an alternative 12-month time horizon analysis was undertaken. In part due to funding cessation, 12-month follow-up data were only available for approximately half of the participants, and cost, outcome and covariate data were imputed for the participants who were not followed up to 12 months. Second, the number of therapy sessions was reduced from the 12 sessions in the base case as implemented in the trial to alternatives of 10 sessions and 8 sessions over a six-month period assuming maintained QoL, to explore the impact of lowering the intervention costs on the cost-effectiveness result. Next, complete case analysis was implemented assuming data were missing completely at random to assess the impact of missing data on the cost-effectiveness results. Lastly, the number of routine NHS physiotherapist visits was excluded from the total cost addressing the possibility that participants could not differentiate between an NHS or trial physiotherapist when answering the questionnaire.

### Subgroup analysis

Effectiveness of the intervention was differential in four subgroups [18] and therefore cost-effectiveness analyses were conducted in subgroups defined by these four criteria. They are: UPDRS scores, Montreal Cognitive assessment cognitive function scores, presence of freezing symptoms measured by a standardised Freezing of gait questionnaire [33], and number of retrospective falls. The incremental effects across the groups were generated using recycled prediction methods to balance the covariates.

## Results

### Intervention cost

The total cost of the PDSAFE intervention was £650 per participant in the intervention arm (Table 1). The cost of physiotherapists’ time and travel expenses accounted for over 95% of the total intervention cost.

**Table 1:** PDSAFE intervention costs

Item	Unit cost (£)	Quantity	Sum (£)
**Therapists training**			
Trainer (lead therapist)	165 per day	1.75 days	288.75
Room hire	150 per day	2 days	300
Training materials	3.50 per therapist	14 therapists	49
**Therapy sessions**			
Therapist time	43.62 per visit	238 participants * 12	124,578.72
Consumables (clinical notes)	1.20 per visit	238 participants * 12	3427.20
Travel expenses	8 per visit	238 participants * 12	22,848
**Patient equipment during the sessions**			
Printed materials	2 per participant	238 participants	476
CDs	0.08 per participant	238 participants	19.04
Weighted vests	54.16 each	23.8^a^	1289.01
Balance pads	19.41 each	59.5^b^	1154.90
Step counts	11.99 each (6.31^c^)	15.87^d^	100.14
**Total cost (per patient n = 238)**	**649.60**		

Note: a. Approximately 1 in 5 participants used a weighted vest. Each weighted vest was used for two participants for all visits. (238/5)/2 = 23.8

b. Approximately 1 in 2 participants used a balance pad. Each pad was used for two participants for all visits. (238/2)/2 = 59.5

c. Annualised cost over 2 years: K/[(1-(1 + r)^-n^)/r]. K = 11.99, r = 3.5%, *n* = 2

d. Approximately 1 in 15 participants used a step count: 238/15 = 15.87

### Resource use and costs

Over the 6-month period, participants in the intervention group had fewer GP, practice nurse or home care help visits, though none of the differences was statistically significant (Supplementary material 2). The most frequently used social service was home care: on average each participant in the intervention group had 13 visits compared to 30 visits in the control group. Overall, there was no statistically significant difference in the cost of resource use between the groups. The total 6-month cost of service use per patient was £3137 (95%CI £2602, £3673) in the intervention group and £3069 (95% £2621, £3518) in the control group, with a non-statistically significant difference of £68 (95%CI -£634, £770).

### QoL and QALYs

**Fig. 1:**
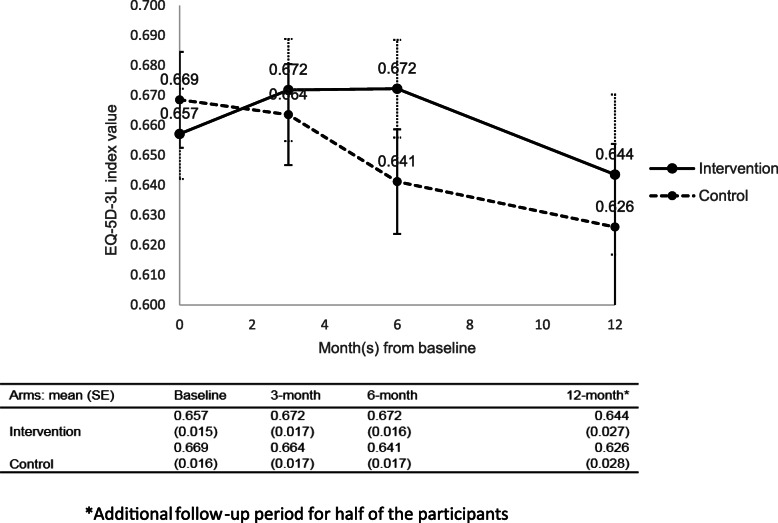
EQ-5D-3L utility values at baseline, 3, 6, and 12 months

The completeness of EQ-5D-3L questionnaire at the month 6 follow-up was 79% (188/238) and 90% (213/236) for the intervention and control groups respectively. The utility values of the intervention group declined slightly less than the control group in the 6-month and 12-month periods (Fig. 1). The differences in utility values between the groups were small (0.031 at 6 months, and 0.017 at 12 months), and not statistically significant.

### Cost-effectiveness base-case analysis

**Fig. 2:**
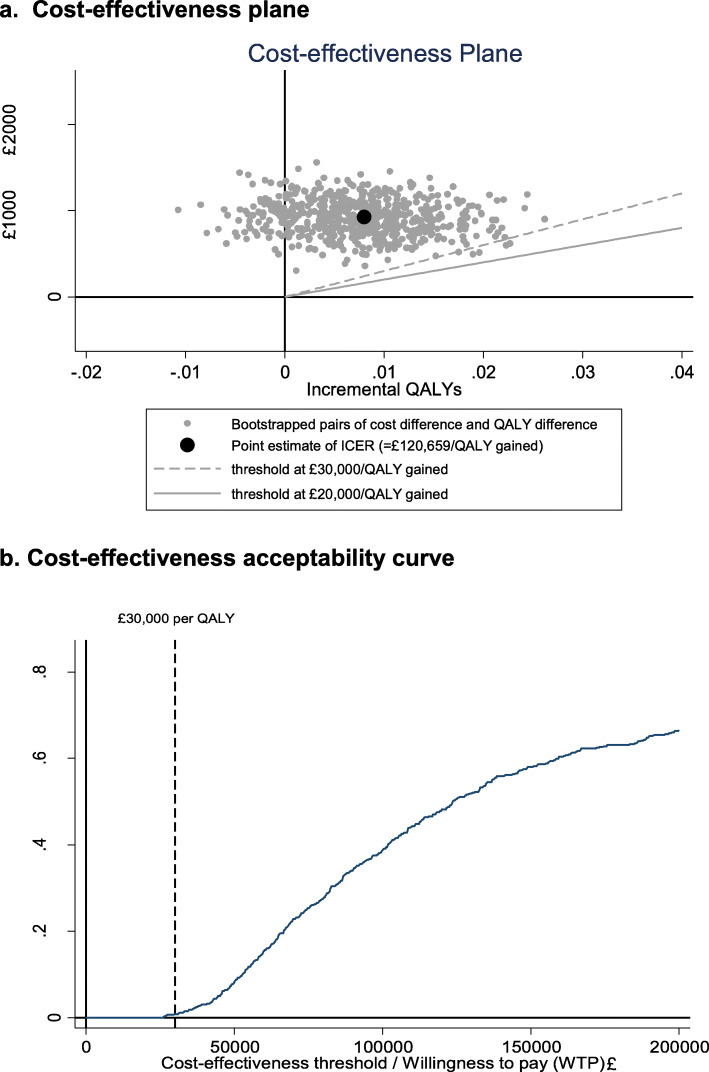
Cost-effectiveness plane (**a**) and cost-effectiveness acceptability curve (**b**) of the PDSAFE intervention vs. usual care

The adjusted costs of the intervention and control group were £4020 (95%CI £3531, £4510) and £3095 (95%CI £2694, £3496) respectively, leading to an incremental cost of £925 (95%CI £428, £1422). The adjusted QALYs of the intervention and control group were 0.336 (95%CI 0.326, 0.345) and 0.328 (95%CI 0.319, 0.337) respectively, which resulted an incremental QALY of 0.008 (95%CI -0.006, 0.021). The resulting ICER was £120,659 per QALY gained. All the bootstrapped cost-utility pairs (Fig. 2a) were in the northern quadrants, indicating that the cost consumed in the intervention group was always greater than in the control group. Similarly, the majority of the simulated cost-utility dyads were in the north-east quadrant, indicating that the intervention was more likely to improve mean health outcomes than the control group. The cost-effectiveness plane crossing the y axis indicates that there was a higher degree of uncertainty surrounding the estimate of the incremental QALY than the estimate of incremental cost, leading to a wide 95% confidence interval for the estimate of ICER. The cost-effectiveness acceptability curve (Fig. 2b) shows that the probability that the intervention was cost-effective at the £30,000 threshold was 0.5%.

### Subgroup and sensitivity analysis

The ICER was above £30,000 for all subgroups indicating the intervention was not cost-effective in any subgroup (Supplementary material Table 3a). The incremental QALYs in the intervention arm for those who had not experienced freezing episodes was statistically significantly greater (0.021, 95% CI 0.0007, 0.041), although the ICER remained high (=£61,687 per QALY). In the sensitivity analyses, while the PDSAFE intervention was still not cost-effective compared to the control arm in all scenarios, the ICER decreased by more than half when estimated over a 12-month time horizon (Supplementary material Table 3b).

## Discussion

The results showed that the PDSAFE intervention was unlikely to be cost-effective from an NHS and PSS’s perspective over the trial horizon. There was minimal difference between arms in the total cost of NHS and social service use. The utility values in the intervention group declined slightly less than the control group over the trial period, but the difference was minimal and not statistically significant.

A high degree of uncertainty over the QALYs gained led to a wide confidence interval in the ICER, which may be due to the small mean QALY gained and large variation among the participants. This may also be related to the limitation of the EQ-5D-3L instrument [34] in capturing the impact of the PDSAFE intervention on QoL aspects such as improved falls efficacy and less fear of falling as identified in the effectiveness results and a qualitative investigation as part of the PDSAFE trial [25].

Sensitivity analyses indicated that the intervention was more likely to be cost-effective if a 12-month time horizon was adopted. If the difference lasts beyond the 12-month follow-up point, the probability of the intervention being cost-effective would be expected to increase because the cost of intervention would be recouped over a longer time horizon and incremental QALY gain would be larger, however further research would be needed to fully examine this. Exploratory analysis of the observed 6 month data found the treatment effect to be greater among the participants who did not provide 12-month data compared to those who did. This suggests that if those participants were followed up to 12 months, a potentially greater treatment effect at 12 months might have emerged. This may also suggest that the participants who received the intervention later may gain more benefit than the participants who received the intervention early, possibly due to the learning curve of the trial physiotherapists, however these could all be due to chance.

Several studies have evaluated the cost-effectiveness of exercise programmes or physiotherapy programmes previously and the results were mixed. Clarke et al. (2016) evaluated effectiveness and cost-effectiveness of physiotherapy and occupational therapy versus no therapy in the PD REHAB randomised controlled trial among 762 patients with mild to moderate Parkinson’s disease [35]. They found that individualised physiotherapy and occupational therapy did not have immediate (3 months) or long-term (15 months) clinically meaningful improvements in activities of daily living or QoL measured by PDQ-39. Although no difference in primary outcome was found, the ICER was only £3493/QALY (−£169,371 to £176,358) with 50.5% probability to be under the £20,000 threshold. The authors, noting the wide confidence interval surrounding the ICER as well as the insignificant QALY gain, concluded that the cost-effectiveness result was contingent on a clinically meaningful primary outcome which had not been found. An earlier trial (2012) by Fletcher et al. reported a more positive result in favour of a 10-week exercise programme with 0.03 (− 0.02 to 0.08) incremental QALY gained and £35 less health and social care costs, which suggested the intervention was dominant with approximately 80% probability that the intervention was cost-effective [36]. The intervention cost £76 per participant for the complete 10-week group sessions in community settings in this trial. Notably, the cost-effectiveness analysis alongside this trial incorporated only 93 of the 130 participants who were randomised, and there was no imputation strategy for missing data hence these results could be subject to selection bias. An Australian study evaluated the cost-effectiveness of a similar exercise intervention led by a physical therapist (a mixture of group and individual sessions) among Parkinson’s population and estimated the cost to be $A574 per fall averted and a ICER of $A338,800/QALY with a 0.005 QALY gain over a 6-month period [37]. The intervention cost was estimated to be $A1,010 (in 2012 Australian dollars, equivalent to £559) per participant which incorporated monthly group exercise classes, and 2 to 4 home visits from the therapist over 6 months. They concluded that the intervention was cost-effective when using a threshold of $A2,000 per fall prevented however the ICER estimate was far over the UK equivalent threshold £20,000 to £30,000 per QALY gained. Compared to the current study, both Clarke et al. and Fletcher et al. [35, 36] reported much more positive cost-effectiveness estimates favouring the intervention, due to the lower intervention costs and higher QALYs gained. The result from the Australian study is consistent with the current study, reporting similar intervention cost (£559, vs. £649 for PDSAFE) and similar QALY (0.005 vs. 0.008 for PDSAFE) gained. Overall the QALY gained in the three earlier studies and the PDSAFE trial were small and measured with substantial uncertainty. Further studies should be conducted to better understand the treatment effect of physiotherapy and its impact on QALY.

## Conclusions

Over the 6-month period, the PDSAFE intervention was associated with a small, statistically insignificant mean QALY gain with large uncertainty, leading to a large ICER with a wide confidence interval. The uncertainty analysis revealed that the probability of the intervention being cost-effective was 0.5% suggesting that the PDSAFE intervention is unlikely to be cost-effective from the NHS and PSS perspective. Sensitivity analysis showed that even if the number of PDSAFE physiotherapy sessions were reduced from 12 to 8, the ICER would still be three times higher than the current NICE threshold due to the unchanged incremental QALY gain. Therefore, cost reduction strategies only, such as changing home-based to group sessions, are unlikely to result in the intervention becoming cost-effective unless effectiveness also improves. The 12-month analysis, however, suggested that the intervention may become cost-effective were the effect to be sustained beyond the intervention period however this would require a study with longer follow-up beyond 12 months.

## Supplementary information

**Additional file 1.** Table 1 Unit costs for health-care resource use. Supplementary material 2 - NHS and social care resource use per patient over 6-month. Supplementary material 3 Subgroup (a) and sensitivity (b) analyses results (over six-month).

## Data Availability

The anonymised datasets used and/or analysed during the current study are available from the corresponding author on reasonable request.

## Abbreviations

CI: Confidence interval
QALY: Quality-adjusted life-years
QoL: Quality of life
ICER: Incremental cost-effectiveness ratio
NHS: National Health Service
PSS: Personal Social Service
