# Supplementary material for: Cost-effectiveness of the PDSAFE personalised physiotherapy intervention for fall prevention in Parkinson’s: an economic evaluation alongside a randomised controlled trial
Source: BMC Neurol. 2020 Aug 11;20:295. doi: 10.1186/s12883-020-01852-8 (PMC7418432; doi:10.1186/s12883-020-01852-8)
Supplement: Supplementary file 1 — Additional file 1. Table 1 Unit costs for health-care resource use. Supplementary material 2 - NHS and social care resource use per patient over 6-month. Supplementary material 3 Subgroup (a) and sensitivity (b) analyses results (over six-month). [file 12883_2020_1852_MOESM1_ESM.docx]

**Supplementary material 1**

**Table 1 - Unit costs for health-care resource use**

| Resource use item (unit used in the source) | Unit Cost | Source |
| --- | --- | --- |
| NHS resource use |  |  |
| GP  (per contact) | £36.00 | PSSRU 2015/16 pg. 145. Per patient contact lasting 9.22 minutes, with qualifications (1) |
| Practice Nurse (per hour) | £43.00  (£11.11 per contact) | PSSRU 2015/16 pg. 143. Nursing average cost per hour, with qualifications (1)  Duration of contact per patient is 15.5 minutes (PSSRU 2014/15, pg.174, based on the 2006/07 UK general practice survey (2) |
| Parkinson’s nurse (per hour) | £75.00  (£19.63 per contact after inflation^a^) | PSSRU 2014/15 pg. 172. Cost per hour of patient-related work as nurse specialist (community) with qualifications. (1)  Duration of contact per patient is 15.5 minutes (PSSRU 2014/15, pg.174, based on the 2006/07 UK general practice survey (2) |
| Health visitor (per contact) | £54 (£54.72 after inflation^a^ ) | PSSRU 2014/15 pg. 171. Cost per face-to-face contact in health visiting services. (1) |
| Social Worker (per hour) | £79.00 | PSSRU 2015/16 pg. 156. Cost per hour of client-related work with qualification. Assume average contact is one hour. (1) |
| NHS physiotherapist (per contact) | £49.00 | NHS reference cost 2015/16. Allied Health professionals (AHP), physiotherapist, adult, one to one (A08A1). National average unit cost (3) |
| Occupational Therapist  (per contact) | £79.00 | NHS reference cost 2015/16. Allied Health professionals (AHP), occupational therapist, adult, one to one (A06A1). National average unit cost (3) |
| Speech or language therapist  (per contact) | £88.00 | NHS reference cost 2015/16. Allied Health professionals (AHP), speech and language therapist, adult, one to one (A13A1). National average unit cost (3) |
| A day case at hospital  (per case) | £733 | Reference costs 2015/16 pg. 10. Table 2: Unit costs by point of delivery, 2013-14 to 2015-16. Unit costs per finished consultant episode (a patient spends in the care of one consultant) for a day case. (3) |
| Hospital Outpatient attendance  (per attendance) | £117 | Reference costs 2015/16 pg. 10. Table 2: Unit costs by point of delivery, 2013/14 to 2015/16. Unit cost per outpatient attendance. (3) |
| A&E by ambulance  (per incidence) | £236 | Reference costs 2015/16 pg. 18. Table 8: Costs by currency for ambulance services between 2013-14 and 2015-16. Unit cost per ‘see and treat and convey’. (3) |
| Ambulance followed by emergency care only (i.e. paramedics, but not convey to hospital)  (per incidence) | £181 | Reference costs 2015/16 pg. 18. Table 8: Costs by currency for ambulance services between 2013-14 and 2015-16. Unit cost per ‘see and treat or refer’. (3) |
| A&E by own/public transport  (per incidence) | £138 | NHS Reference costs 2015/16 pg. 10. Table 2: Table 2: Unit costs by point of delivery, 2013-14 to 2015-16. Unit cost per A&E attendance. (3) |
| Hospital Stay for treatment  (per day) | £373.00 | NHS reference costs 2015/16 main schedule. Average of cost per elective and non-elective inpatient excess bed days across all currency codes. Elective inpatient excess bed days, average across all currency codes: £395. Non-elective inpatient excess bed days, average across all currency codes: £351. (3) |
| Respite care at hospital  (per day) | £264.75 | NHS reference costs main schedule 2015-2016. Estimated from the day cost for respite care with length of stay 4 days or less (£1059/4=£264.75) (3) |
| Social care resource use | |  |
| Home care/ home help  (per hour) | £30.75 (£15.375 per contact) | PSSRU 2015/16 pg. 29. 1.5 Home care for older people. Average standard hourly rate for services provided in-house. Assumed 30 min per contact. (1) |
| Meals on wheels  (per time) | £3.00 | South Lanarkshire council service, social care and health, meals at home (meals on wheels) (4) |
| Day centre  (per attendance) | £61 | PSSRU 2015/16 pg. 28. 1.4 Local authority own-provision day care for older people. Unit cost per client attendance (1) |
| Lunch club  (per time) | £3.09 | Glasgow city council, social care and health, adults and older people, a lunch club (5) |
| Sitting service (per hour) | £21 | PSSRU 2015/16 pg.85. 6.12 Short-break provision for disabled children and their families. Unit cost per family per hour. Assumed one hour per contact (1) |
| Night Care  (per hour) | £31.83 | PSSRU 2015/16 pg. 160. 11.6 Home care worker. Applied price multipliers for unsocial hours: 1.035 for an independent sector home care hour. Assumed one hour per contact. (1) |

a. HCHS inflation factor 1.013 (2014/15 Pay and prices index (PPI) 293.1 / 2015/16 PPI 297.0)

Reference:

(1) Curtis L & Burns A. Unit Costs of Health and Social Care. PSSRU, University of Kent. 2016.

(2) Information Centre. 2006/07 UK general practice workload survey. Primary Care Statistics, Information Centre, Leeds. 2007.

(3) Department of Health. NHS reference cost 2015 / 16. 2016. Available from: https://www.gov.uk/government/publications/nhs-reference-costs-2015-to-2016.

Access date: 22 June 2020

(4) South Lanarkshire council service. Social care and health, meals at home (meals on wheels) (2017). Available from: ttp://www.southlanarkshire.gov.uk/info/200227/care_for_the_elderly/485/meals_at_home_meals_on_wheels. Accessed on 05 August 2018

(5) Glasgow city council. social care and health, adults and older people, a lunch club. 2017. Available from: https://www.glasgow.gov.uk/index.aspx?articleid=17293. Accessed on 05 August 2018

**Supplementary material 2 - NHS and social care resource use per patient over 6-month**

| Resource use  (No. of visits) | Intervention (N=238) | | | | | |  | Control (N=236) | | | | | |  | p value |
| --- | --- | --- | --- | --- | --- | --- | --- | --- | --- | --- | --- | --- | --- | --- | --- |
|  | N | Mean | SD | Median | Min | Max |  | N | Mean | SD | Median | Min | Max |  |  |
| NHS |  |  |  |  |  |  |  |  |  |  |  |  |  |  |  |
| GP | 144 | 2.72 | 2.47 | 2 | 0 | 16 |  | 177 | 3.23 | 2.80 | 3 | 0 | 15 |  | 0.09 |
| Practice nurse | 140 | 1.39 | 1.67 | 1 | 0 | 9 |  | 170 | 2.09 | 4.67 | 1 | 0 | 43 |  | 0.09 |
| PD nurse | 138 | 1.21 | 1.45 | 1 | 0 | 11 |  | 177 | 1.47 | 1.67 | 1 | 0 | 11 |  | 0.15 |
| Health visitor | 138 | 0.11 | 0.54 | 0 | 0 | 5 |  | 170 | 0.17 | 0.76 | 0 | 0 | 8 |  | 0.42 |
| Social worker | 136 | 0.07 | 0.38 | 0 | 0 | 3 |  | 170 | 0.17 | 0.72 | 0 | 0 | 7 |  | 0.16 |
| NHS physiotherapist | 136 | 2.48 | 3.64 | 0 | 0 | 16 |  | 170 | 1.34 | 2.42 | 0 | 0 | 14 |  | 0.00 |
| Occupational therapist | 136 | 0.35 | 0.93 | 0 | 0 | 6 |  | 169 | 0.50 | 1.44 | 0 | 0 | 12 |  | 0.29 |
| Speech therapist | 135 | 0.89 | 2.09 | 0 | 0 | 11 |  | 167 | 0.59 | 2.03 | 0 | 0 | 21 |  | 0.22 |
| Day hospital | 137 | 0.93 | 1.89 | 0 | 0 | 14 |  | 170 | 0.90 | 1.57 | 0 | 0 | 10 |  | 0.89 |
| Outpatient clinic | 142 | 1.78 | 2.09 | 1 | 0 | 13 |  | 173 | 1.78 | 1.79 | 1 | 0 | 13 |  | 1.00 |
| A&E by ambulance | 137 | 0.14 | 0.42 | 0 | 0 | 2 |  | 167 | 0.11 | 0.33 | 0 | 0 | 2 |  | 0.48 |
| Ambulance with paramedics only | 131 | 0.26 | 0.83 | 0 | 0 | 6 |  | 153 | 0.19 | 0.63 | 0 | 0 | 5 |  | 0.42 |
| A&E by own transport | 135 | 0.16 | 0.45 | 0 | 0 | 3 |  | 165 | 0.22 | 0.59 | 0 | 0 | 4 |  | 0.31 |
| Hospitalisation for treatment | 187 | 0.30 | 2.51 | 0 | 0 | 30 |  | 208 | 0.07 | 0.47 | 0 | 0 | 5 |  | 0.19 |
| Hospitalisation for respite care | 187 | 0 | 0 | 0 | 0 | 0 |  | 208 | 0 | 0 | 0 | 0 | 0 |  | - |
|  |  |  |  |  |  |  |  |  |  |  |  |  |  |  |  |
| Social care |  |  |  |  |  |  |  |  |  |  |  |  |  |  |  |
| Home care / home help | 181 | 13.43 | 50.55 | 0 | 0 | 390 |  | 192 | 29.99 | 103.30 | 0 | 0 | 910 |  | 0.05 |
| Meals on wheels | 187 | 1.04 | 12.49 | 0 | 0 | 169 |  | 207 | 1.00 | 12.77 | 0 | 0 | 182 |  | 0.98 |
| Day center | 186 | 0.49 | 4.35 | 0 | 0 | 52 |  | 206 | 0.63 | 4.59 | 0 | 0 | 52 |  | 0.75 |
| Luncheon club | 186 | 0.21 | 2.86 | 0 | 0 | 39 |  | 204 | 0.51 | 6.43 | 0 | 0 | 91 |  | 0.56 |
| Sitting service | 186 | 0.91 | 7.16 | 0 | 0 | 65 |  | 206 | 1.33 | 7.75 | 0 | 0 | 78 |  | 0.58 |
| Night care | 186 | 0.98 | 13.34 | 0 | 0 | 182 |  | 206 | 0.06 | 0.91 | 0 | 0 | 13 |  | 0.33 |

**Supplementary material 3 Subgroup (a) and sensitivity (b) analyses results (over six-month)**

| Analysis | Incremental cost  (intervention – control) (95%CI) (£) | Incremental QALYs (intervention – control)  (95%CI) | ICER (£/QALY gained) |  |
| --- | --- | --- | --- | --- |
| Base-case | 925 (428, 1422) | 0.008 (-0.006, 0.021) | 120,659 |  |
| **a. Subgroup analyses** |  |  |  |  |
| **Subgroup by MOCA (cognitive function)** | | | | |
| Impaired (MOCA<=25) (n=184) | 293 (-890, 1476) | 0.007 (-0.014, 0.029) | 39,486 |  |
| Not impaired (25<MOCA<28) (n=122) | 1513 (207, 2819) | 0.018 (-0.008, 0.043) | 84,925 |  |
| Normal cognitive function (MOCA>=28) (n=168) | 1514 (329, 2698) | 0.0006 (-0.020, 0.021) | 2,613,629 |  |
| **Subgroup by the presence of freezing symptoms** | | | | |
| Non-freezer (n=182) | 1285 (234, 2335) | 0.021 (0.0007, 0.041) | 61,687 |  |
| Freezer (n=291) | 852 (-170, 1873) | -0.0006 (-0.018, 0.017) | Control dominates |  |
| **Subgroup by UPDRS (Parkinson’s severity)** | | | | |
| Lowest disease severity (UPDRS<= 22) (n=153) | 1956 (747, 3166) | 0.006 (-0.015, 0.028) | 301,605 |  |
| Moderate disease severity (22<UPDRS<39) (n=161) | 500 (-761, 1761) | 0.016 (-0.006, 0.039) | 30,731 |  |
| Highest disease severity (UPDRS>=39) (n=158) | 600 (-640, 1840) | -0.004 (-0.027, 0.019) | Control dominates |  |
| **Subgroup by retrospective falls** | | | | |
| 1 fall (n=99) | 1201 (-117, 2520) | 0.010 (-0.018, 0.038) | 197,810 |  |
| 2-3 falls (n=198) | 1121 (7, 2235) | 0.009 (-0.011, 0.030) | 53,860 |  |
| >3 falls (n=177) | 725 (-532, 1981) | 0.007 (-0.014, 0.029) | 82,532 |  |
|  |  |  |  |  |
| **b. Sensitivity analyses** |  |  |  |  |
| **Time horizon:** 12-month (base case: 6-month) | 1176 (249, 2104) | 0.021 (-0.011, 0.054) | 55,176 |  |
| **Cost:** 10 sessions (actual number of sessions) (base case: 12 sessions) | 825 (328, 1322) | 0.008 (-0.005, 0.021) | 105,360 |  |
| **Cost:** No. of sessions reduced to 8 (base case: 12 sessions) | 719 (226, 1213) | 0.008 (-0.005, 0.021) | 91,876 |  |
| **Missing data handling:** Complete case analysis (n=288) (base case: multiple imputation) | 1139 (588, 1689) | 0.010 (-0.006, 0.025) | 117,226 |  |
| **Routine use of physiotherapist:** Routine physiotherapist visit not included in the overall cost | 903 (416, 1390) | 0.008 (-0.005, 0.020) | 120,086 |  |
